# Serum Osteoprotegerin Levels and the Vascular Reactivity Index in Patients with Hypertension

**DOI:** 10.3390/medicina59101794

**Published:** 2023-10-09

**Authors:** Yen-Liang Chen, Po-Yu Huang, Jen-Pi Tsai, Ji-Hung Wang, Bang-Gee Hsu

**Affiliations:** 1 Department of Internal Medicine, Hualien Tzu Chi Hospital, Buddhist Tzu Chi Medical Foundation, Hualien 97004, Taiwan; a0975797460@yahoo.com.tw; 2 Division of Nephrology, Department of Internal Medicine, Dalin Tzu Chi Hospital, Buddhist Tzu Chi Medical Foundation, Chiayi 62247, Taiwan; poyuhs13628@gmail.com (P.-Y.H.); tsaininimd1491@gmail.com (J.-P.T.); 3 School of Medicine, Tzu Chi University, Hualien 97004, Taiwan; 4 Division of Cardiology, Hualien Tzu Chi Hospital, Buddhist Tzu Chi Medical Foundation, Hualien 97004, Taiwan; 5 Division of Nephrology, Hualien Tzu Chi Hospital, Buddhist Tzu Chi Medical Foundation, Hualien 97004, Taiwan

**Keywords:** endothelial dysfunction, hypertension, osteoprotegerin, vascular reactivity index

## Abstract

*Background and Objectives*: Osteoprotegerin (OPG), a soluble glycoprotein found in serum, has been associated with both the presence and severity of atherosclerosis. OPG is regarded as the mediator in the process of vascular endothelial dysfunction. Impaired endothelial function has an intimate link with hypertension (HTN) and is associated with significant morbidity and mortality. This study was to investigate the connection between OPG and endothelial dysfunction in patients having HTN. *Materials and Methods*: There are 102 patients with HTN included. For the purpose of determining the levels of OPG, a commercial enzyme-linked immunosorbent test kit was applied. The vascular reactivity index (VRI), which is assessed via the digital thermal monitoring, provides information on endothelial function. *Results*: Ten patients with HTN (9.8%) were classified as having poor vascular reactivity (VRI < 1.0), 46 HTN patients (45.1%) as having intermediate vascular reactivity (1.0 ≤ VRI < 2.0), and 46 HTN patients (45.1%) were classified as having high vascular reactivity (VRI ≥ 2.0). A greater serum OPG level (*p* < 0.001) and older age (*p* = 0.022) were linked to impaired vascular reactivity. The estimated glomerular filtration rate (*r* = 0.196, *p* = 0.048) was positively correlated with VRI values in hypertensive participants, while advanced age (*r* = −0.222, *p* = 0.025) and the log-transformed OPG level (log-OPG, *r* = −0.357, *p* < 0.001) were negatively correlated with VRI. Serum log-OPG level was shown to be strongly and independently correlated with VRI values in HTN individuals after multivariable forward stepwise linear regression analysis (β = −0.357, adjusted R^2^ change = 0.119, *p* < 0.001). *Conclusions*: In patients with HTN, serum OPG levels were adversely correlated with VRI and probably had a role in endothelial dysfunction.

## 1. Introduction

The presence of hypertension (HTN) gives rise to adverse cardiovascular (CV) sequelae. The hazard ratio (HR) for major CV diseases (CVDs) was 0.36 [95% confidence interval (CI), 0.26–0.51] and the HR for all-cause mortality was 0.47 (95% CI, 0.32–0.67), according to data from a systemic analysis that compared non-hypertensive candidates with hypertensive patients with systolic blood pressure (SBP) ≥ 160 mm Hg [1]. Endothelial dysfunction plays a contributing role in developing HTN via imbalances in endogenous vasoactive molecules, induction of inflammatory response, oxidative stress, and aberrant activation of renin-angiotensin-aldosterone system [2,3,4]. Undiagnosed and untreated endothelial dysfunction and related vasculopathy can substantially increase the risk of serious arterial complications including stenosis and aneurysm formation [5].

The measurement of endothelial function has changed over time. In 1986, a research using coronary angiography showed that atherosclerotic coronary artery disease (CAD) also affects its function, causing exaggerated vasoconstriction as a result of endothelial dysfunction [6]. While the historical “gold standard” remains the estimation of defective endothelial-dependent dilation during coronary angiographic procedures, several noninvasive measures have been devised, such as brachial artery diameter via non-invasive ultrasound imaging [6]. Digital thermal monitoring (DTM), a novel noninvasive, automated, and operator-independent method, has been created to assess endothelial function. The vascular reactivity index (VRI), the result of DTM, has been demonstrated to be a useful physiological marker for endothelial function [7].

A growing body of research indicates that endothelial dysfunction can be used as a marker for atherosclerosis before structural changes to the arterial wall are visible on angiography or ultrasonography [8]. These initiatives cover a wide range of activities, such as verification of plasma biomarkers that reflect pathogenic occurrences within developing lesions (e.g., release of cytokines associated with inflammation, such as interleukin-1 (IL-1) and IL-6, or loss of inducible luminal surface adhesion molecules, such as vascular cell adhesion protein-1 (VCAM-1)), as well as the markers of systemic inflammatory status such as C-reactive protein [9].

Osteoprotegerin (OPG), one of the members of the tumor necrosis factor (TNF) receptor superfamily, is also called osteoclastogenesis inhibitory factor. Besides its protective effect on bone resorption, OPG mediates vascular calcification and atherogenesis [10]. Mice lacking OPG showed calcified lesions of the media in aorta and renal arteries, indicating that OPG appears to defend against vascular calcification, according to an experimental study [11,12]. Serum OPG level has a significant and independent predictive value for CV risk in osteoporotic patients, and plasma OPG level is strongly associated with endothelial function [13]. Additional research supports an elevated OPG to the receptor activator of nuclear factor kappa B ligand (RANKL) ratio as a potential indicator of endothelial dysfunction progression in autoimmune disorders; the alteration of OPG to RANKL proportion is considered to have therapeutic implications [14].

The relationship between the level of OPG and endothelial function in patients with HTN, a recognized risk factor for CVD, has not been studied, though. The purpose of this research was to assess how the biomarker OPG is related to the physiological endothelial function test, or VRI, in patients with HTN.

## 2. Materials and Methods

### 2.1. Patients

Between July 2016 and April 2017, 102 HTN patients who visited the outpatient CV clinic of the Buddhist Tzu Chi General Hospital in Hualien, Taiwan were enrolled. Hualien Tzu Chi Hospital, Buddhist Tzu Chi Medical Foundation, and the Research Ethics Committee all gave their approval for this research. Patients with >50% stenosis in any segment after coronary angiography were determined to have CAD after their medical data were reviewed. All study participants had their morning blood pressure readings taken on the right arm using standard mercury sphygmomanometers with proper cuff sizes after taking a rest for more than 10 min. SBP and diastolic blood pressure (DBP) were averaged after being taken three times at 5-min intervals. The definition of HTN was in accordance with the Eighth Joint National Committee (JNC 8). Patients were identified as having diabetes mellitus (DM) when the fasting plasma glucose concentrations were 126 mg/dL or higher for at least twice, or if they were taking oral antidiabetic drugs or receiving insulin treatment. Prior to the study, a signed informed consent form was required from each participant. Participants who were not able to give their informed consent or who have active infectious diseases, acute myocardial infarction, malignancy, amputation, or heart failure at the time of blood collection were excluded from the study.

### 2.2. Anthropometric Analysis

After overnight fast, anthropometric factors were evaluated the following morning. The closest 0.5 kg and 0.5 cm were used to determine body weight and height, respectively. The body mass index was determined by dividing weight (kg) by height cubed (m^2^).

### 2.3. Biochemical Investigations

After fasting for a night, blood samples were taken. In order to perform biochemical analyses, 5 mL of blood from each patient was promptly centrifuged at 3000× *g* for 10 min and stored at 4 °C. We measured serum levels of total calcium, phosphorus, fasting glucose, albumin, blood urea nitrogen (BUN), creatinine, total cholesterol (TCH), triglycerides, high-density lipoprotein cholesterol (HDL-C), and low-density lipoprotein cholesterol (LDL-C) using an autoanalyzer (Siemens Advia 1800; Siemens Healthcare, Henkestr, Erlangen, Germany). Serum OPG levels were determined using commercially available enzyme-linked immunosorbent tests (eBioscience Inc., San Diego, CA, USA) [15]. Using the equation developed by the Chronic Kidney Disease Epidemiology Collaboration, the estimated glomerular filtration rate (eGFR) was determined.

### 2.4. Endothelial Function Measurements

We used a DTM device authorized by the US Food and Drug Administration to collect endothelial function parameters from patients who had fasted overnight and avoided using caffeine, alcohol, tobacco, and other stimulants, as well as vasoactive drugs (VENDYS-II; Endothelix, Inc., Houston, TX, USA). The participants were kept in a supine position and relaxed for 30 min in a room with temperature set between 22 and 24 °C. Afterwards, blood pressure cuffs and skin temperature sensors were placed on the patients’ right upper arms and both index fingers (left, control finger; right, finger undergoing occlusion). During the 5-min stabilization, 5-min cuff inflation, and 5-min deflation phases, DTM of both wrists was done. A reactive hyperemic response was induced in the fingertip distally by quickly inflating the right upper arm cuff for five minutes to a pressure of 50 mm Hg higher than the SBP and then rapidly deflating it. The greater the vascular reactivity, the more the temperature rebounded. As determined by the VENDYS software (https://www.vendys2.com/), VRI is the maximum temperature differential between the rebound curve and the zero reactivity curves during the reactive hyperemia period. Poor vascular reactivity was indicated by a VRI of less than 1.0, intermediate vascular reactivity was indicated by a VRI of 1.0 to 1.9, and good vascular reactivity was indicated by a VRI of 2.0 or higher [7].

### 2.5. Statistical Analysis

The Kolmogorov-Smirnov test was adopted for the determination of normal distribution of the continuous variables, which were calculated as means ± standard deviation (SD). For parameters with non-normal distribution (fasting glucose, TCH, triglycerides, BUN, creatinine, and OPG), a Kruskal-Wallis analysis was used to compare measured values between groups (divided into those with poor, intermediate, and good VRI). For parameters with normal distribution, a one-way analysis of variance was used. To achieve normality, fasting glucose, TCH, triglycerides, BUN, creatinine, and OPG levels were logarithmically transformed prior to subsequent analysis. Simple linear regression analyses were used to investigate variables that were correlated with VRI, and significant variables were incorporated into multivariable forward stepwise regression analysis. Univariable and multivariable logistic regression analyses were used to find out the correlates of vascular reactivity dysfunction and poor vascular reactivity. After confirming the effect of OPG level on vascular reactivity dysfunction and poor vascular reactivity, we depicted the receiver operating curve (ROC) to determine the power by the area under the curve (AUC). The achievement of statistical significance was defined as *p*-values of less than 0.05. The SPSS for Windows was used to perform all the analytic processes of the statistics (Version 19.0; SPSS Inc., Chicago, IL, USA).

## 3. Results

Table 1 displays the baseline characteristics and antihypertensive medications taken by the complete cohort of 102 hypertensive patients. Ten (9.8%) of the 102 HTN patients had poor VRI, 46 (45.1%) had intermediate VRI and 46 (45.1%) had good VRI. Among the comorbid diseases, 52 patients (51.0%) had DM, 70 (68.6%) had coronary artery disease, and 18 patients (17.6%) had a history of smoking. Among the participants, the poor VRI group had significantly older age (*p* = 0.022) and higher OPG levels (*p* < 0.001). The following factors were not significantly different between the groups: gender, smoking history, presence of DM or coronary artery disease, and use of antihypertensive medications or anti-lipid medications.

**Figure 1:**
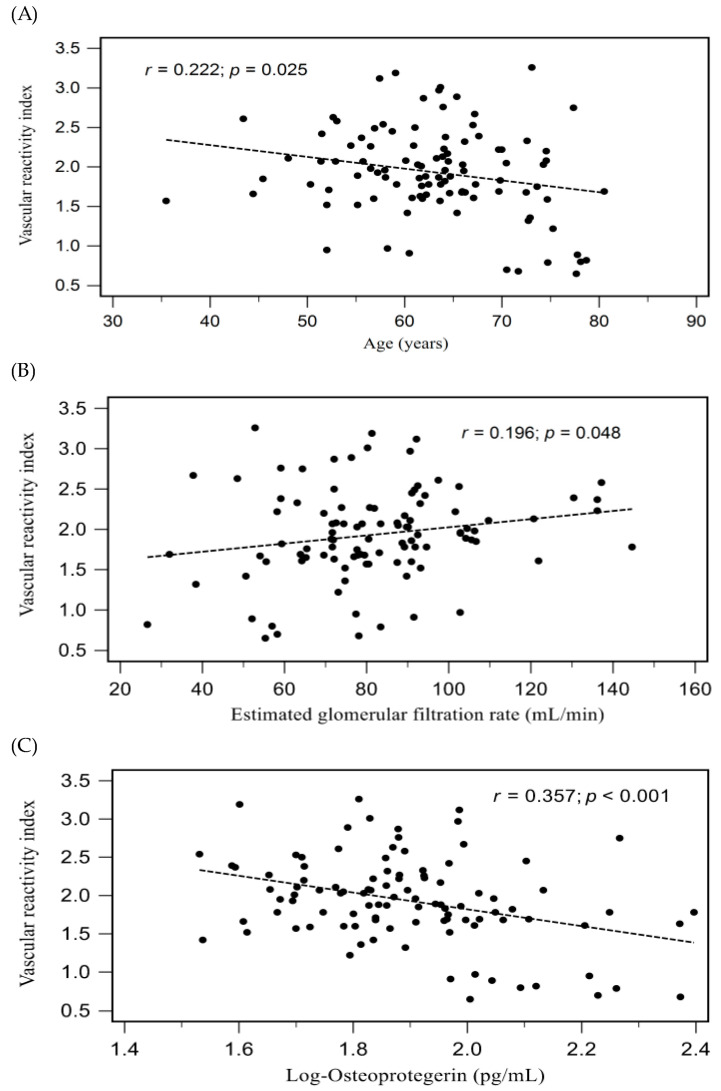
Relationships between VRI and (**A**) age, (**B**) eGFR, and (**C**) log-transformed OPG (log-OPG) among patients with hypertension.

For the 102 HTN individuals, Table 2 shows the correlation between clinical characteristics and serum VRIs. It was discovered that eGFR levels (*r* = 0.196, *p* = 0.048) were favorably correlated with VRI values, while advanced age (*r* = −0.222, *p* = 0.025) and serum log-transformed OPG levels (log-OPG, *r* = −0.357, *p* < 0.001) were negatively correlated with VRI values. A low serum level of log-OPG (β = 0.357, *p* < 0.001) was significantly and independently associated with VRI in hypertensive individuals, according to a stepwise forward linear regression analysis of the variables that were significantly associated with VRIs. Figure 1A–C showed two dimensional scattered plots of VRI values with age, eGFR level, and serum log-OPG level among hypertensive patients, respectively.

Serum OPG was independently associated with vascular reactivity dysfunction (odds ratio (OR) = 1.025; 95% confidence interval (CI) = 1.008–1.041; *p* = 0.003) and poor vascular reactivity index (OR = 1.028, 95% CI = 1.011–1.045, *p* = 0.001) in HTN patients by logistic regression analysis after being adjusted for certain factors (*p* < 0.2) of poor vascular reactivity in Table 1, including sex, age, BMI, and eGFR (Table 3). The ROC curve for predicting vascular reactivity dysfunction by OPG revealed that the AUC was 0.679 (95% CI = 0.575–0.783, *p* < 0.001) and poor vascular reactivity by OPG demonstrated the AUC was 0.905 (95% CI = 0.843–0.968, *p* < 0.001), respectively (Table 4). Furthermore, Table S1 listed the correlation of ongoing use of different antihypertensives or lipid-lowering agents with serum OPG levels. We found that whether different classes of drugs were being used or not did not have significant association with OPG.

## 4. Discussion

The major findings of the present study were that in hypertensive patients, serum OPG concentrations, in addition to advancing age and worse kidney function, were an independent correlate with endothelial dysfunction measured with VRI.

Genetic predisposition, environmental factors such as air pollution, and preexisting CV risk factors all make patients become more vulnerable to endothelial dysfunction [16]. Endothelial dysfunction is associated with initiation and progression of a number of molecular pathways, including altered metabolism of nitric oxide (NO), oxidative stress, expression of adhesion molecules and pro-coagulant factors, introduction of pro-inflammatory cytokines, induction of apoptosis, and epithelial–mesenchymal transition [17,18]. As a consequence, endothelial dysfunction is the “gateway” to atherosclerotic CVD. It has been suggested that endothelial function serves as a “barometer” of CV risk. Since the emergence of biochemical markers and noninvasive physiological measurements, studies have attempted to link these parameters. Poor VRI was significantly correlated with both a higher Framingham risk score and a higher myocardial calcium score, based on a prior investigation focusing on asymptomatic adults [19]. VRI can provide more information about an individual’s state of endothelial function, while more research is required to integrate such physiologic measurements into clinical practice.

As mentioned previously, OPG, produced by both the CV system and the bone, has the ability to control both osteoclastogenesis and vascular calcification [10,11,20]. In endothelial cells, OPG is involved in several biological pathways. OPG binds directly to RANKL, interfering with its interaction with the RANK receptor on the endothelium and thereby regulating vascular calcification. Furthermore, OPG can act as a receptor for the TNF-related apoptosis-inducing ligand (TRAIL), inhibiting the effects of TRAIL on the up-regulation of endothelial NO synthase and down-regulation of reactive oxygen species production. In addition, OPG activates the renin-angiotensin system and induces the expression of vascular endothelial growth factors, with the resultant inflammatory and fibrotic processes. Most of these mechanisms can contribute to endothelial dysfunction [14,21]. In summary, OPG contributes to changes in endothelial function through multiple yet not fully understood pathways.

The present study discovered that OPG levels were independently associated with VRI among HTN patients. Some studies have investigated the relationship between OPG and endothelial dysfunction. HTN patients were found to have higher serum OPG concentrations than those without high blood pressures; patients with a higher risk for CVD or with target organ damage had significantly higher OPG levels [22,23]. In patients with chronic kidney disease (CKD), there was a significant correlation between SBP variability and blood OPG concentrations; the relationship was more obvious between subgroups having less comorbidities and those without DM [24]. A number of studies have identified a significant association between OPG and endothelial dysfunction, with the study groups recruiting patients with type 2 DM, hyperuricemia, and Hashimoto’s thyroiditis in euthyroid state; in these studies, flow-mediated dilation of the brachial artery via ultrasonography was used for the estimation of endothelial function [25,26,27]. However, several studies investigating diabetic patients and women with polycystic ovary syndrome did not reveal OPG levels as a correlate of altered endothelial function [23,28]. Patients on dialysis had significantly higher blood OPG levels than the control group, regardless of serum parathyroid hormone levels [29]. As kidney function deteriorated in patients with CKD and on maintenance hemodialysis, serum OPG gradually increased and was positively correlated with inflammatory markers, but most critically was linked to survival [30,31,32,33]. According to a meta-analysis, there was a 1.04 higher chance of CV mortality in CKD patients (including those who received dialysis) whenever the concentration of OPG increased by 1 pmol/L [34]. These studies collectively indicated that OPG could modulate endothelial pathophysiology and could even have an effect on patient survival.

Lower eGFR was found to be significantly correlated with higher VRI values; in other words, there was an association between kidney failure and endothelial dysfunction. The pathophysiology of endothelial dysfunction in CKD includes an inflammatory response, oxidative stress, accumulation of advanced glycation end products, positive phosphorus balance with subsequent increase in fibroblast growth factor 23 and deficient active vitamin D, and an increase in certain uremic toxin levels, including asymmetric dimethylarginine and symmetric dimethylarginine, both inhibitors of endothelial NO synthase, which thus limit the availability of NO [35,36,37]. Our previous research revealed that in patients with CKD, both indoxyl sulfate concentrations and DBP were inversely correlated with VRI [38]; however, in the present study, patients with greater VRIs did not have significantly higher DBPs. A cross-sectional study investigated the endothelial function in patients with non-dialysis CKD, by determining reactive hyperemia index through the measurement of peripheral arterial tonometry; the indices did not increase with advancing stages of CKD or the amount of proteinuria [39].

Our research revealed that age was negatively correlated with VRI levels, but increasing age was not an independent predictor of endothelial dysfunction. The association was attenuated probably due to the presence of CV risk factors and comorbid conditions [40]. Aging causes oxidative stress and increased production of reactive oxygen species, metabolic derangements, and dysregulated angiogenesis; these factors result in a propensity to endothelial senescence [41].

We have to admit that there are several study limitations. The study was cross-sectional and involved a limited number of patients. Even though we can identify a connection between VRI and OPG, we are unable to determine whether OPG is a factor that protects or harms endothelial cells, nor can we say whether OPG has any causal relationships with endothelial dysfunction. Further studies with a prospective and longitudinal design are therefore mandated. Furthermore, certain inflammatory parameters such as C-reactive protein were not measured in the study, which might provide more information about the pathophysiology of endothelial dysfunction and its relationship with OPG in patients with HTN.

## 5. Conclusions

In this research, there was a negative correlation between VRI and serum OPG in HTN patients. These results suggested that OPG might serve as a biomarker and modulator in the control of endothelial dysfunction, but further investigations on the population-specific design are required for confirmation.

## Figures and Tables

**Table 1:** Clinical characteristics according to different vascular reactivity index by digital thermal monitoring.

Characteristics	All Participants (*n* = 102)	Good Vascular Reactivity (*n* = 46)	Inermediate Vascular Reactivity (*n* = 46)	Poor Vascular Reactivity (*n* = 10)	*p* Value
Age (years)	63.01 ± 8.45	62.24 ± 7.55	62.27 ± 8.53	69.98 ± 9.65	0.022 *
Height (cm)	163.84 ± 7.46	162.50 ± 7.85	165.57 ± 7.38	162.00 ± 3.97	0.100
Body weight (kg)	73.31 ± 11.73	72.81 ± 10.19	75.39 ± 13.30	66.07 ± 7.75	0.068
Body mass index (kg/m^2^)	27.27 ± 3.70	27.61 ± 3.74	27.40 ± 3.76	25.16 ± 2.70	0.158
Vascular reactivity index	1.93 ± 0.57	2.41 ± 0.35	1.70 ± 0.18	0.82 ± 0.11	<0.001 *
SBP (mmHg)	135.75 ± 18.67	136.52 ± 16.92	125.91 ± 20.42	131.40 ± 19.29	0.735
DBP (mmHg)	80.23 ± 10.88	81.59 ± 9.39	79.54 ± 12.05	77.10 ± 11.77	0.426
Total cholesterol (mg/dL)	158.00 (139.75–178.00)	152.50 (139.50–178.00)	158.00 (138.50–175.75)	167.50 (142.50–189.50)	0.657
Triglyceride (mg/dL)	141.00 (103.50–212.75)	147.50 (105.50–220.75)	131.50 (97.25–204.00)	179.50 (83.75–217.00)	0.678
HDL-C (mg/dL)	46.13 ± 10.47	46.23 ± 10.39	45.61 ± 10.16	48.00 ± 12.97	0.806
LDL-C (mg/dL)	89.68 ± 27.99	87.98 ± 30.76	92.07 ± 26.71	86.50 ± 20.91	0.732
Fasting glucose (mg/dL)	113.00 (95.75–154.25)	115.00 (95.75–155.50)	111.00 (96.50–165.25)	107.50 (87.25–144.25)	0.619
Albumin (mg/dL)	4.37 ± 0.23	4.40 ± 0.25	4.35 ± 0.17	4.35 ± 0.32	0.521
Blood urea nitrogen (mg/dL)	17.00 (13.75–19.25)	15.50 (13.00–18.25)	17.00 (14.00–22.00)	18.00 (13.25–23.00)	0.099
Creatinine (mg/dL)	1.00 (0.80–1.10)	0.90 (0.78–1.10)	1.00 (0.90–1.10)	1.00 (0.96–1.40)	0.127
eGFR (mL/min)	81.81 ± 21.91	85.77 ± 22.32	80.81 ± 20.51	68.24 ± 22.43	0.065
Osteoprotegerin (pg/mL)	76.69 (60.48–98.72)	68.03 (51.78–83.64)	79.51 (62.92–100.17)	127.86 (102.63–172.47)	<0.001 *
Male, *n* (%)	83 (81.4)	34 (73.9)	42 (91.3)	7 (70.0)	0.063
Diabetes mellitus, *n* (%)	52 (51.0)	26 (56.5)	21 (45.7)	5 (50.0)	0.579
Coronary artery disease, *n* (%)	70 (68.6)	31 (67.4)	34 (73.9)	5 (50.0)	0.326
Smoking, *n* (%)	18 (17.6)	11 (23.9)	5 (10.9)	2 (20.0)	0.255
ACE inhibitor use, *n* (%)	20 (19.6)	8 (17.4)	11 (23.9)	1 (10.0)	0.530
ARB use, *n* (%)	49 (48.0)	25 (54.3)	18 (39.1)	6 (60.0)	0.250
β-blocker use, *n* (%)	47 (46.1)	21 (45.7)	21 (45.7)	5 (50.0)	0.966
CCB use, *n* (%)	48 (47.1)	25 (54.3)	19 (41.3)	4 (40.0)	0.408
Statin use, *n* (%)	77 (75.5)	33 (71.7)	36 (78.3)	8 (80.0)	0.722
Fibrate use, *n* (%)	7 (6.9)	4 (8.7)	2 (4.3)	1 (10.0)	0.653

Values for continuous variables given as means ± standard deviation and test by one-way analysis of variance; variables not normally distributed given as medians and interquartile range and test by Kruskal-Wallis analysis; values are presented as number (%) and analysis after analysis by the chi-square test. SBP, systolic blood pressure; DBP, diastolic blood pressure; HDL-C, high-density lipoprotein cholesterol; LDL-C, low-density lipoprotein cholesterol; eGFR, estimated glomerular filtration rate; ACE, angiotensin-converting enzyme; ARB, angiotensin-receptor blocker; CCB, calcium-channel blocker. * *p* < 0.05 was considered statistically significant.

**Table 2:** Correlation of vascular reactivity index levels and clinical variables by simple or multivariable linear regression analyses.

Variables	Vascular Reactivity Index				
	Simple Regression		Multivariable Regression		
	*r*	*p* Value	Beta	Adjusted R^2^ Change	*p* Value
Male	0.032	0.746	—	—	—
Diabetes mellitus	0.099	0.320	—	—	—
Coronary artery disease	0.168	0.091	—	—	—
Smoking	0.124	0.213	—	—	—
Age (years)	−0.222	0.025 *	—	—	—
Height (cm)	−0.061	0.545	—	—	—
Body weight (kg)	0.004	0.971	—	—	—
Body mass index (kg/m^2^)	0.057	0.570	—	—	—
Systolic blood pressure (mmHg)	0.075	0.454	—	—	—
Diastolic blood pressure (mmHg)	0.141	0.158	—	—	—
Log-TCH (mg/dL)	−0.096	0.335	—	—	—
Log-Triglyceride (mg/dL)	0.041	0.680	—	—	—
HDL-C (mg/dL)	−0.006	0.955	—	—	—
LDL-C (mg/dL)	−0.027	0.790	—	—	—
Log-Glucose (mg/dL)	0.093	0.352	—	—	—
Albumin (mg/dL)	0.138	0.168	—	—	—
Log-BUN (mg/dL)	−0.189	0.057	—	—	—
Log-Creatinine (mg/dL)	−0.190	0.056	—	—	—
eGFR (mL/min)	0.196	0.048 *	—	—	—
Log-Osteoprotegerin (pg/mL)	−0.357	<0.001 *	−0.357	0.119	<0.001 *

Data of TCH, triglyceride, fasting glucose, blood urea nitrogen, creatinine, and osteoprotegerin showed skewed distribution and, therefore, were log-transformed before analysis. Analysis of data was done using the simple linear regression analyses or multivariable stepwise linear regression analysis (adapted factors were age, eGFR, and log-osteoprotegerin). TCH, total cholesterol; HDL-C, high-density lipoprotein cholesterol; LDL-C, low-density lipoprotein cholesterol; BUN, blood urea nitrogen; eGFR, estimated glomerular filtration rate. * *p* < 0.05 was considered statistically significant.

**Table 3:** Univariable and multivariable logistic regression analysis for vascular reactivity dysfunction (intermediate vascular reactivity and poor vascular reactivity) or poor vascular reactivity.

Model	Osteoprotegerin (per 1 pg/mL of Increase) for Vascular Reactivity Dysfunction		Osteoprotegerin (per 1 pg/mL of Increase) for Poor Vascular Reactivity	
	OR (95% CI)	*p* Value	OR (95% CI)	*p* Value
Crude model	1.020 (1.006–1.034)	0.005 *	1.024 (1.010–1.038)	0.001 *
Adjusted model	1.025 (1.005–1.041)	0.003 *	1.028 (1.011–1.045)	0.001 *

Adjusted model: sex, age, body mass index, and estimated glomerular filtration rate. OR—odds ratio; CI—confidence interval. * *p* < 0.05 was considered statistically significant.

**Table 4:** Diagnostic value of osteoprotegerin on vascular reactivity dysfunction (intermediate vascular reactivity and poor vascular reactivity) or poor vascular reactivity.

**Vascular Reactivity** **Dysfunction**						
	**AUC (95% CI)**	**Cut-off**	**Sen (%)**	**Spe (%)**	**PPV (%)**	**NPV (%)**
**Osteoprotegerin (pg/mL)**	0.679 (0.575–0.783)	89.72	51.8	82.6	78.4	58.5
**Poor Vascular Reactivity**						
	**AUC (95% CI)**	**Cut-off**	**Sen (%)**	**Spe (%)**	**PPV (%)**	**NPV (%)**
**Osteoprotegerin (pg/mL)**	0.905 (0.843–0.968)	93.11	100.0	78.3	33.4	100.0

AUC, area under the curve; 95% CI, 95% confidence interval; Sen, sensitivity; Spe, specificity; PPV, positive predictive value; NPV, negative predictive value. All *p* < 0.001.

## Data Availability

Upon request, the corresponding author can provide the data utilized in this study.

## Supplementary Materials

The following supporting information can be downloaded at: https://www.mdpi.com/article/10.3390/medicina59101794/s1, Table S1: Correlation of ongoing different antihypertensive drugs or lipid-lowering drugs with serum osteoprotegerin concentrations among study patients with hypertension.
