# Supplementary material for: Serum Osteoprotegerin Levels and the Vascular Reactivity Index in Patients with Hypertension
Source: Medicina (Kaunas). 2023 Oct 9;59(10):1794. doi: 10.3390/medicina59101794 (PMC10608475; doi:10.3390/medicina59101794)
Supplement: Supplementary file 1 [file medicina-59-01794-s001.zip › medicina-2603536-supplementary.pdf]

Table S1. Correlation of ongoing different antihypertensive drugs or lipid-lowering drugs with serum osteoprotegerin concentrations among study patients with hypertension.

**Drugs use characteristics (*n* = 102)**	**Osteoprotegerin (pg/mL)**	***p* value**
No ACE inhibitor use (*n* = 82)	77.79 (61.74-99.28)	0.453
ACE inhibitor use (*n* = 20)	69.48 (57.68-93.82)	
No ARB use (*n* = 53)	76.04 (58.58-105.15)	0.841
ARB use (*n* = 49)	74.07 (63.15-94.43)	
No β-blocker use (*n* = 55)	71.90 (51.75-93.36)	0.081
β-blocker use (*n* = 47)	81.13 (67.30-103.06)	
No CCB use (*n* = 54)	72.17 (55.05-102.83)	0.500
CCB use (*n* = 48)	78.22 (66.53-97.07)	
No statin use (*n* = 25)	81.26 (67.39-103.79)	0.261
Statin use (*n* = 77)	72.20 (59.32-97.67)	
No fibrate use (*n* = 95)	75.73 (61.01-98.25)	0.620
Fibrate use (*n* = 7)	67.50 (60.16-96.36)	

Values not normally distributed are given as median and interquartile range and tested by Mann–Whitney U test; ACE, angiotensin converting enzyme; ARB, angiotensin receptor blocker; CCB, calcium channel blocker.
